# APOMAB®, a La-Specific Monoclonal Antibody, Detects the Apoptotic Tumor Response to Life-Prolonging and DNA-Damaging Chemotherapy

**DOI:** 10.1371/journal.pone.0004558

**Published:** 2009-02-27

**Authors:** Fares Al-Ejeh, Jocelyn M. Darby, Chris Tsopelas, Douglas Smyth, Jim Manavis, Michael P. Brown

**Affiliations:** 1 Experimental Therapeutics Laboratory, Hanson Institute, Adelaide, South Australia, Australia; 2 Department of Nuclear Medicine, Royal Adelaide Hospital, Adelaide, South Australia, Australia; 3 Centre for Neurological Disease, Hanson Institute, Adelaide, South Australia, Australia; 4 Department of Medical Oncology, Royal Adelaide Hospital Cancer Centre and School of Medicine, The University of Adelaide, Adelaide, South Australia, Australia; Genentech, United States of America

## Abstract

**Background:**

Antineoplastic therapy may impair the survival of malignant cells to produce cell death. Consequently, direct measurement of tumor cell death *in vivo* is a highly desirable component of therapy response monitoring. We have previously shown that APOMAB® representing the DAB4 clone of a La/SSB-specific murine monoclonal autoantibody is a malignant cell-death ligand, which accumulates preferentially in tumors in an antigen-specific and dose-dependent manner after DNA-damaging chemotherapy. Here, we aim to image tumor uptake of APOMAB® (DAB4) and to define its biological correlates.

**Methodology/Principal Findings:**

Brisk tumor cell apoptosis is induced in the syngeneic EL4 lymphoma model after treatment of tumor-bearing mice with DNA-damaging cyclophosphamide/etoposide chemotherapy. Tumor and normal organ accumulation of Indium 111 (^111^In)-labeled La-specific DAB4 mAb as whole IgG or IgG fragments was quantified by whole-body static imaging and organ assay in tumor-bearing mice. Immunohistochemical measurements of tumor caspase-3 activation and PARP-1 cleavage, which are indicators of early and late apoptosis, respectively, were correlated with tumor accumulation of DAB4. Increased tumor accumulation of DAB4 was associated directly with both the extent of chemotherapy-induced tumor cell death and DAB4 binding per dead tumor cell. Tumor DAB4 accumulation correlated with cumulative caspase-3 activation and PARP-1 cleavage as tumor biomarkers of apoptosis and was directly related to the extended median survival time of tumor-bearing mice.

**Conclusions/Significance:**

Radiolabeled La-specific monoclonal antibody, DAB4, detected dead tumor cells after chemotherapy, rather than chemosensitive normal tissues of gut and bone marrow. DAB4 identified late apoptotic tumor cells in vivo. Hence, radiolabeled DAB4 may usefully image responses to human carcinoma therapy because DAB4 would capture the protracted cell death of carcinoma. We believe that the ability of radiolabeled DAB4 to rapidly assess the apoptotic tumor response and, consequently, to potentially predict extended survival justifies its future clinical development as a radioimmunoscintigraphic agent.

This article is part I of a two-part series providing proof-of-concept for the the diagnostic and therapeutic use of a La-specific monoclonal antibody, the DAB4 clone of which is represented by the registered trademark, APOMAB®.

## Introduction

Neoplasia results from an imbalance between rates of cellular proliferation and survival in a tissue [1]. Successful antineoplastic treatment controls tumor growth by inhibiting cellular proliferation and/or survival. Ideally, precise multi-parametric measures of cellular proliferation and survival in vivo may enable patient outcomes to be determined earlier than conventional measures allow [2].

Most patients with metastatic malignancy are not curable, and may be treated with systemic cytotoxic chemotherapy to palliate cancer-related symptoms and/or to prolong life. Most cytotoxic regimens comprise DNA-damaging drugs, and tumor response rates are generally less than 50%. To know if chemotherapy is working, patients are usually scanned after two or three cycles (or six to nine weeks) of treatment with computed tomography (CT) to assess lesion size by Response Evaluation Criteria in Solid Tumors (RECIST). Apart from evident clinical improvement, CT evidence of lack of progression may indicate that treatment is effective and may be used in the decision to continue treatment. To minimize patient exposure to potentially toxic and ineffective treatment, therapy monitoring technologies are being developed [3]–[6]. Among these technologies, ^18^F-fluorodeoxyglucose-positron emission tomography (FDG-PET) is closest to widespread clinical acceptance [2], [7], [8]. However, it should be noted that FDG uptake in vivo is non-specific and is a composite measure of several biological processes. For example, FDG may be taken up by inflammatory as well as neoplastic tissues [9]. Furthermore, loss of tumor uptake of FDG within hours of treatment of gastro-intestinal stromal tumor with imatinib [10] or of non-small lung cancer with gefitinib [11] represents reduced glucose utilization rather than tumor cell death [9]. In some malignancies, tumor uptake of FDG may best be considered as a measure of cell viability rather than as a direct measure of cell death or of the proliferative status of tumor cells.

An unmet medical need exists for robust, minimally invasive, and universally applicable measures of early tumor response to anti-cancer treatment. Molecular imaging methods, in particular, may yield personalized and real-time assays of biologically significant processes such as apoptosis. Fusion of PET images with CT or magnetic resonance images provides precise and quantitative data, and delivers three-dimensional representations of the essential anatomic and pathophysiologic heterogeneity of many tumors [2], [12]. Importantly, commercial imperatives are driving the use of therapy monitoring technologies to streamline “go/no go” decision making in the protracted and costly exercise of drug development [13]. As stated recently: “Real-time imaging of cell death would be a coveted application with which to assess the efficacy of cytotoxic drugs and, potentially, to monitor the toxicity of these drugs in normal tissues” [14].

Radiolabeled recombinant annexin V, which binds phosphatidylserine everted on the exterior surface of apoptotic cells, has been investigated extensively as an in vivo marker of apoptosis [15]–[17], although annexin V also detects necrotic cells [18]. However, the small size and short biologic half-life of annexin V limit its utility as a marker of therapy response. Moreover, earlier clinical development of radiolabeled annexin V (as Apomate®) was stalled because a one-to-one correspondence between ^99m^Tc- 6-hydrazinopyridine-3-carboxylic acid-(HYNIC)-annexin V uptake and radiologic response to cancer chemotherapy was not found [19].

Notwithstanding the importance of apoptosis as a mode of cancer cell death [20], the response of carcinomas to antineoplastic treatment commonly takes days to weeks and may derive significant contributions from other processes such as necrosis, autophagy, mitotic catastrophe, and cell senescence [21], [22]. Irrespective of the pathway to tumor cell death, dead cells remain a common feature of many malignancies [23], [24], and may increase in number after primary chemotherapy [25], [26] despite evasion of apoptosis being recognized as a hallmark of cancer [1]. Recently, the view has emerged that defective ‘waste disposal’, which is the phagocytic clearance of dead cells, contributes to the generation of autoantibodies including those with La/SSB specificity in systemic autoimmune diseases [27]. Similarly, inefficient and/or saturated clearance mechanisms may produce in tumours an excess of dead cells, which have lost cell membrane integrity allowing purposeful entry of diagnostic or therapeutic antibodies [28].

Recently, we discovered that a La-specific monoclonal antibody (mAb) behaved as a malignancy-associated cell death ligand that preferentially binds apoptotic malignant cells [29]. During apoptosis, the La antigen translocates from nucleus to cytoplasm [30], and is fixed in dying cells by transglutaminase 2 (TG2) [29]. As cell membrane integrity is lost during the late phase of apoptosis, cytoplasmic La becomes accessible to binding by specific mAb, which in turn becomes crosslinked in the dying cell by TG2 so that its levels are higher than in dead cells of the counterpart primary cell type [29]. Studies in EL4 tumor-bearing mice show that, after cytotoxic chemotherapy, binding of this La-specific mAb to tumors was specific and saturable with low accumulation in normal organs [31]. The La-specific DAB4 clone, which is represented by the APOMAB® trademark, originated from a murine autoantibody [32]. The antigen-binding domain of DAB4 has been cloned, sequenced, expressed, and found to bind with low nanomolar affinity to a La epitope, which is highly conserved between humans and rodents (Al-Ejeh et al., unpublished data).

Selective in vivo targeting of La favors two major applications of this technology in cancer diagnosis and treatment. First, La-directed radioimmunoscintigraphy may detect early tumor responses to antineoplastic treatment particularly with DNA-damaging drugs and ionizing radiation. Second, La-directed radioimmunotherapy may act by radiation-induced bystander killing of neighboring tumor cells and may be enhanced by radiosensitizing drugs. Here, we describe in vivo studies in which we use radioimmunoscintigraphy to show that tumor accumulation of an optimally prepared immunoconjugate of the La-specific mAb, DAB4 [33], correlates not only with biomarkers of tumor cell apoptosis but also with extended survival of tumor-bearing mice after escalated doses of chemotherapy.

## Methods

### Ethics statement

The Animal Ethics Committee of the Institute of Medical and Veterinary Sciences gave approval for use of the mice. In the use and care of the mice, we followed the humane research principles of replacement, reduction and refinement endorsed by the National Health and Medical Research Council of Australia. Replacement was not possible because there were no alternative techniques to tumor-bearing mice. We achieved a reduction in the numbers of animals used through improved experimental design. Refinement of procedures to improve the welfare of the animals, such as use of analgesics, avoiding significant adverse effects, and enhanced housing conditions, were adopted.

### Antibody production, purification and radiolabeling

Protein-G-purified murine mAb from cultures of La-specific DAB4 (IgG2aκ) or control Sal5 (isotype-matched) hybridomas [29], [31] were conjugated to 1,4,7,10-tetraazacyclododedane-*N*,*N*′,*N*″,*N*‴-tetraacetic acid mono-(N-hydroxysuccinimidyl) (DOTA-NHS) ester and radiolabeled with Indium-111 (^111^In) [31], [33]. Radioactivity was measured using the Cobra 5010 gamma counter (PerkinElmer Inc., Wellesley, MA), which was normalized for Indium-111 counting using a 100–350 keV counting window. F(ab)_2_ fragments were prepared using agarose-immobilized pepsin according to the manufacturer's instructions (Pierce, IL).

### EL4 lymphoma model

We adopted the well-characterized EL4 lymphoma model of apoptosis induction [34] with modifications as described [31]. Briefly, 10^6^ EL4 cells were inoculated subcutaneously in the right flank of 6–8 week-old syngeneic C57BL/6 (B6) mice.

### Biodistribution studies

C57BL/6 mice with 1 week-old EL4 tumor implants were untreated or given 25 mg/kg cyclophosphamide and 19 mg/kg etoposide (full-dose chemotherapy) by intraperitoneal injection (IPI). ^111^In-DOTA-DAB4 was given by intravenous injection (IVI) via the tail vein to tumor-bearing mice with or 24 h after chemotherapy, and an organ assay was performed [31]. Briefly, radioactivity in counts per minute (cpm) of harvested organs was divided by the mass to give cpm/g. Then, organ accumulation was expressed as the injected dose per gram (%ID/g) of tissue, which was calculated as the percentage of mass-normalized counts to the total counts (cpm) of ^111^In-DOTA-DAB4 at time 0.

### Scintigraphic imaging of EL4 tumors

^111^In-DOTA-DAB4 was administered i.v. (24 h after chemotherapy) to each of the following groups of mice: no chemotherapy - control mice with 4 day-old EL4 tumor implants; half dose chemotherapy - mice with 5 day-old EL4 tumor implants; and full dose chemotherapy - 7 day-old EL4 tumor implants. This protocol allowed for comparison of animals with size-matched tumors, a methodology that mimics clinical practice. Mice were euthanized by asphyxiation following anesthetic overdose. At 48 h after radioligand injection, whole body static images were acquired for 18 min. using a scintigraphic gamma camera (Starcam 300 M; GE, USA). After imaging, organ assays were done [31]. The same procedure was used for ^111^In-labeled F(ab)_2_ fragments in separate experiments, except that D-lysine (40 mg/mL saline) was administered 30 min. before radiotracer injection, and then every 1 h thereafter for a total of 5 injections [35].

### Survival analysis

EL4-tumor bearing mice were grouped as untreated control, or treated with half-dose or full-dose chemotherapy. Bidimensional tumor measurements using calipers were made every second day, and tumor volume was calculated using the formula: largest diameter×[shortest diameter]^2^/2. Mice were euthanized by cervical dislocation when tumors reached 500 mm^3^. Kaplan-Meier plots were used to derive the median survival time using software (GraphPad Prism v.4.0).

### Ex vivo studies on EL4 tumors

Tumors were finely minced with scissors into pieces <10 mm^3^, and a weighed proportion (0.1 g) of tumor mince was suspended in collagenase Type 1 in Hanks' Buffered Salt Solution (HBSS) (10 mL; 2 mg/mL) containing 2.5 mM Ca^2+^. Suspensions were incubated at 37°C with constant rotation for 1 h. Digested tumor cell suspensions were passed sequentially through a series of needles (in order of 19G, 23G, and 25G) to remove coarse materials, and then the last filtrate was centrifuged at 350×*g* for 5 min. Pellets were washed with HBSS (10 mL), resuspended in HBSS (1 mL), and aliquots (100 µL) stained for 30 min. at room temperature (RT) in duplicate with either Sal5 or DAB4 (5 µg/mL). Cells were washed twice with phosphate buffered saline (PBS), and incubated with rabbit anti-mouse IgG Alexa_488_ antibody (2 µg/mL) for 30 min. at RT in the dark. Cells were washed thrice with PBS, resuspended in 7-Amino-Actinomycin D (7-AAD) (2 µg/mL), and analyzed 10 min. later using a FACScan (BD Biosciences, San Jose, CA).

Apoptosis was detected in tumors by immunohistochemical analysis of caspase-3 activation and cleavage of poly ADP-ribose-1 (PARP-1). EL4 tumor-bearing mice were either untreated (control) or treated with half- or full-dose chemotherapy. At 0, 24, 48 and 72 h, tumors were fixed in 10% formalin in PBS, bisected, then each half was embedded in paraffin and sectioned. Sections were stained with hematoxylin and eosin (H&E) or rabbit IgG raised against either activated caspase-3 (1 µg/mL, Chemicon-Millipore, MA) or cleaved PARP-1 (1∶100 of stock, Promega, WI), and subsequently stained with biotin-conjugated anti-rabbit IgG antibody (1 µg/mL; Rockland Inc., PA) followed by streptavidin-horse radish peroxidase (HRP) (1 µg/mL). Entire sections were scanned using the DotSlide acquisition program (Soft Imaging System, Olympus, Tokyo, Japan) on a DotSlide BX51 Olympus light microscope (Olympus) at 20× magnification.

Scanned tumor sections were visualized using OlyVIA (Olympus Viewer for Imaging Applications) software, and images of entire sections or 6 random regions at 10× or 20× were obtained for analysis using analySIS® software (Soft Imaging System, Olympus). Phase color analysis was performed for all images using pixels to define the different phases: viable cells were defined by blue-counterstained nuclei, apoptotic cells were defined by brown staining from 3, 3′-diaminobenzine (DAB) deposition, and necrotic areas were defined as faint blue areas, which lacked appropriate nuclear morphology. For counting of necrotic cells, and activated caspase-3^+^ and cleaved PARP-1^+^ cells, tumors were bisected, and two images were captured from each tumor section to generate a total of 8 images from each tumor. In each case, numbers of necrotic, activated caspase-3^+^ and cleaved PARP-1^+^ cells were divided by the number of viable cells, and then expressed as percentages.

Co-localization studies of tumor-bound DAB4, activated caspase-3 and cleaved PARP-1 were based on immunofluorescence detection. Mice were given 100 µg DAB4-biotin or 100 µg Sal5-biotin (negative control) i.v. 24 h after chemotherapy, and tumors were dissected 24 h later. Tumor-bound DAB4-biotin was detected by confocal microscopy using streptavidin-Alexa_488_ (1 µg/mL). Activated caspase-3 and cleaved PARP-1 were detected using specific rabbit IgG followed by Alexa_546_-conjugated goat anti-rabbit IgG (2.5 µg/mL, Molecular Probes-Invitrogen, CA). Stained tumor sections were mounted using ProLong Gold antifade reagent (Molecular Probes), and slides were analyzed using an Olympus confocal microscope (Bio-Rad; Hercules, CA) with appropriate filters and under constant conditions of laser voltage, iris aperture, and photomultiplier tube amplification.

### Statistical analysis

Statistical analysis was performed with GraphPad Prism v4.0 software. Unless otherwise stated, intergroup comparisons were made by two-way analysis of variance (ANOVA), with p<0.05 being considered significant. Kaplan-Meier median survival curves were compared using log-rank Mantel-Cox test, with p<0.05 being considered significant.

## Results

### Effects of chemotherapy on biodistribution of ^111^In-DOTA-DAB4 in EL4 lymphoma bearing mice, schedule, and antigen specificity

**Figure 1:**
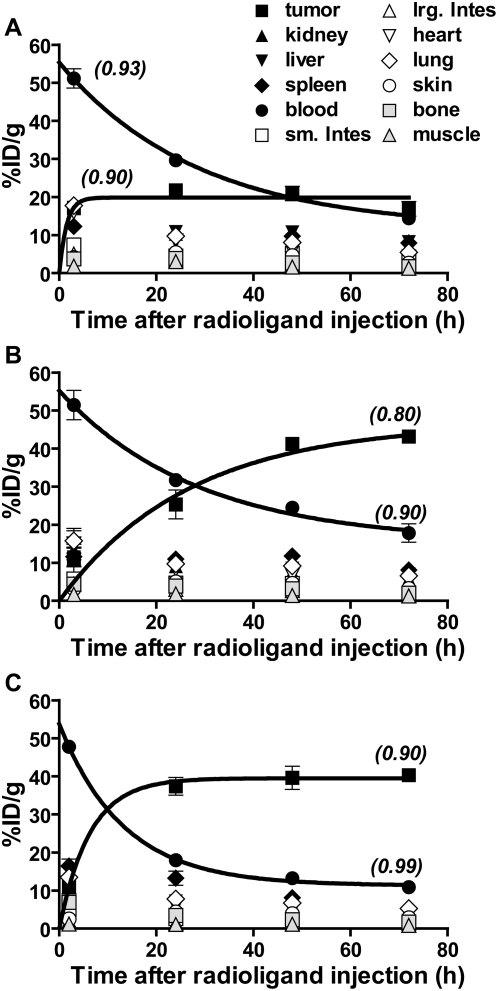
Effects of schedule of radioligand injection on biodistribution of ^111^In-DOTA-DAB4 in mice bearing EL4 lymphoma. Biodistribution of ^111^In-DOTA-DAB4 in EL4 tumor-bearing mice (n = 5/group), which were A, untreated, or B and C, treated with Chemo (19 mg/kg etoposide and 25 mg/kg cyclophosphamide i.p.i.), and given i.v. ^111^In-DOTA-DAB4 A, alone, B, immediately after Chemo, or C, 24 h after Chemo. At 0, 3, 24, 48, and 72 h post-^111^In-DOTA-DAB4, organ assays were used to calculate mean percentage of injected dose per gram of tissue (%ID/g; as described in Methods). Indicated are regression coefficient (*r^2^*) values for curves fitted to clearance and accumulation data using one-phase exponential decay and association models, respectively. Blood clearance and tumor accumulation were accelerated by delaying i.v. ^111^In-DOTA-DAB4 until 24 h post-chemo. Error bars for all graphs; ±SEM.

Previously, we reported antigen-specific and chemotherapy-dependent accumulation in EL4 lymphomas of ^111^In-labeled La-specific mAb, which was injected immediately after chemotherapy [31]. Now, we find that when ^111^In-DOTA-DAB4 was injected 24 hours after chemotherapy, it accumulated more rapidly in EL4 tumors as it cleared more quickly from blood (Figure 1). In EL4 tumor-bearing mice, the blood clearance half-life of ^111^In-DOTA-DAB4 was 19.8±0.2 hours (Figure 1A), which was unchanged (19.8±0.3 hours) by giving ^111^In-DOTA-DAB4 immediately after chemotherapy (Figure 1B). In contrast, giving ^111^In-DOTA-DAB4 24 hours after chemotherapy accelerated its blood clearance with a half-life of 9.2±0.1 hours, and significantly hastened its tumor accumulation with a half-life of 4.1±0.2 hours compared with 18.6±0.3 hours if ^111^In-DOTA-DAB4 was injected immediately after chemotherapy (*P*<0.001) (Figure 1C).

Similarly, administering ^111^In-DOTA-DAB4 immediately after chemotherapy did not alter its uptake in normal organs compared with the same uptake in control mice. In contrast, giving ^111^In-DOTA-DAB4 24 hours after chemotherapy reduced normal organ uptake to levels significantly lower than in control mice (see Figure S1 online).

### Comparisons of tumor accumulation of ^111^In-DOTA-DAB4 with fluorocytometric analysis of tumor cell death and ex vivo tumor cell binding of DAB4

**Figure 2:**
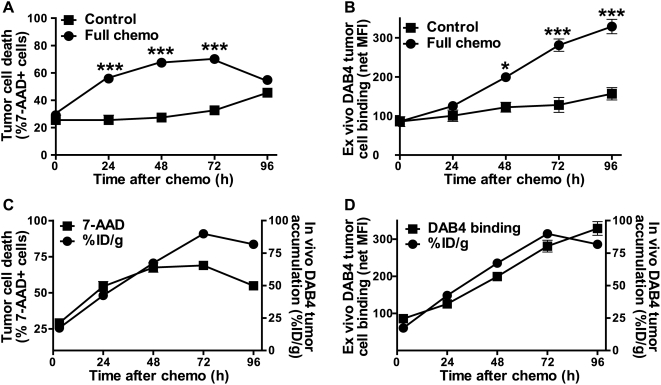
Comparison of cell death, ex vivo and in vivo DAB4 accumulation in EL4 lymphoma model. EL4 tumor-bearing mice were untreated (Control) or treated with 19 mg/kg etoposide and 25 mg/kg cyclophosphamide i.p.i. (Chemo) (n = 3/group). Single cell suspensions were prepared from tumors and stained in duplicate with 7-AAD alone, or with 7-AAD and Sal5 or DAB4. Fluorocytometric analysis revealed levels of tumor cell death (%7-AAD^+^ cells) and of DAB4 binding to 7-AAD^+^ dead tumor cells. A, mean %7-AAD^+^ (±SEM) tumor cells, *** *P*<0.001 versus control. B, La-specific binding to 7-AAD^+^ tumor cells was measured as net mean fluorescence intensity (MFI) (±SEM) of DAB4 binding after subtraction of MFI for Sal5 binding,* *P*<0.05, *** *P*<0.001 versus control. C and D, in a separate study, in vivo tumor accumulation of DAB4 mAb itself (as %ID/g) was calculated after correction for physical radio-decay of Indium-111 and plotted against values either for *C*, %7-AAD^+^ tumor cells or *D*, ex vivo binding of DAB4 to 7-AAD^+^ tumor cells.

To examine the effect of chemotherapy on the frequency of EL4 tumor cell death and the intensity of ex vivo binding of DAB4 to dead tumor cells, we analyzed single tumor cell suspensions, first from control tumors. Here, the frequency of dead 7-AAD^+^ cells increased moderately over 96 hours (Figure 2A) whereas per cell ex vivo binding of DAB4 increased marginally (Figure 2B). In contrast, chemotherapy not only markedly increased the frequency of dead tumor cells (Figure 2A) but also produced a cumulative increase in DAB4 binding to each dead tumor cell (Figure 2B), and suggested that DNA-damaging drugs induced tumor cell expression of La in vivo.

Intratumoral accumulation of the DAB4 mAb itself was determined after correction for the physical decay of Indium-111. In control tumors during the 96-hour observation period, the frequency of dead 7-AAD^+^ cells increased from 26±3 to 46±2% (Figure 2A) in parallel with the increase in DAB4 tumor accumulation from 17±1 to 34±4% of the injected dose per gram of tumor tissue (see Figure S2 online). In contrast, at 72–96 hours after chemotherapy, tumor accumulation of DAB4 reached a plateau at approximately 75–80% of the injected dose per gram of tumor tissue (Figure 2C). However, tumor accumulation of DAB4 tended to track the extent of ex vivo binding of DAB4 (Figure 2D) rather than reflect the frequency of tumor cell death (Figure 2C).

### Immunohistochemical analysis of EL4 tumor caspase-3 activation and PARP-1 cleavage and immunofluorescence analysis of in vivo tumor binding of DAB4

**Figure 3:**
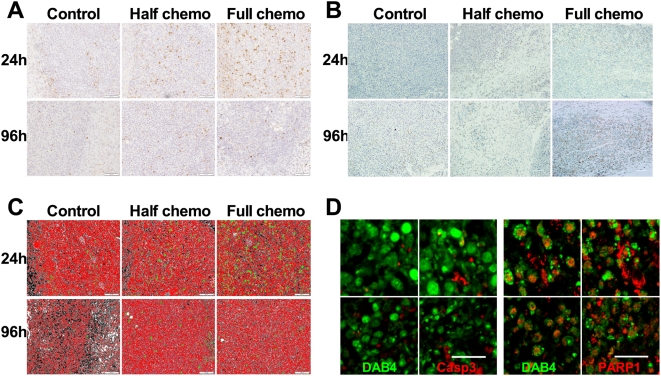
Effects of chemotherapy on caspase-3 activation and PARP-1 cleavage in EL4 tumors. Mice (n = 3/group) were untreated (Control) or treated i.p. with 9.5 mg/kg etoposide and 12.5 mg/kg cyclophosphamide (Half chemo) or 19 mg/kg etoposide and 25 mg/kg cyclophosphamide (Full chemo). Formalin-fixed paraffin-embedded tumor sections were prepared for immunohistochemical detection and phase color analysis. Shown are representative photomicrographs at 24 h or 96 h post-chemo of A, activated caspase-3, and B, cleaved PARP-1. C, shown are representative images of phase color analysis, which are pseudo-colored: viable cells (red); necrotic cells (black); activated caspase-3^+^ (green). D, representative confocal fluorescence microscopy images of EL4 tumor sections from n = 3 mice showing co-staining of DAB4 (green) and (left-hand panels) activated caspase-3 (red) or (right-hand panels) cleaved PARP-1 (red) 24 h after i.v. biotinylated DAB4, which in turn was given 24 h after full chemo. Scale bar, 20 µm. DAB4 localized with late apoptotic cells.

Chemotherapy induced dose-dependent caspase-3 activation and PARP-1 cleavage in tumors of EL4 lymphoma-bearing mice (Figure 3). Immunohistochemical analysis indicated that tumor activation of caspase-3 became most evident 24 hours after a full dose of chemotherapy (Figure 3A) whereas PARP-1 cleavage was best identified 96 hours post-chemotherapy (Figure 3B). Imaging software and phase color analysis were used to quantify these differences (Figure 3C). For example, as control tumors grew over 96 hours, areas of central necrosis (coded black, Figure 3C) expanded in tandem with the increased control tumor content of 7-AAD^+^ cells (Figure 2A). Next, we investigated the physical relationship between stages of apoptosis and the in vivo binding to apoptotic EL4 tumor cells of DAB4, which was antigen specific (data not shown). In this experiment, tumor-bearing mice were given injections of DAB4-biotin 24 hours after chemotherapy, and tumors were harvested for immunofluorescence analysis 24 hours later (i.e. 48 hours after chemotherapy). As illustrated in Figure 3D, DAB4 was observed to be intimately related to cell remnants containing cleaved PARP-1 rather than activated caspase-3, suggesting that DAB4 mainly bound EL4 tumors via its binding of late apoptotic cells.

### Gamma camera imaging of EL4 tumor uptake of ^111^In-DOTA-DAB4 after chemotherapy

**Figure 4:**
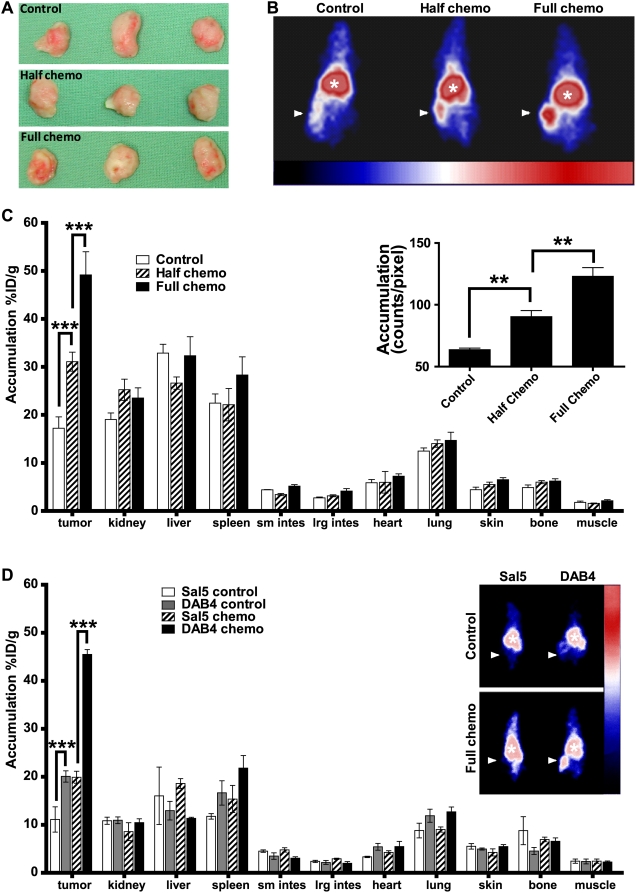
Effects of chemotherapy on gamma camera imaging and biodistribution of ^111^In-DOTA-DAB4 in EL4 lymphoma-bearing mice. A, as described in Methods, growth of EL4 tumors in untreated control mice was staggered so that tumor sizes matched those in mice treated with half- or full-dose chemotherapy. B, representative clinical gamma camera images (arrow, tumor; asterisk, central blood pool) of n = 3 mice. Mice bearing size-matched EL4 tumors were untreated (Control) or given i.v. ^111^In-DOTA-DAB4 24 h after 9.5 mg/kg etoposide and 12.5 mg/kg cyclophosphamide (Half chemo) or 19 mg/kg etoposide and 25 mg/kg cyclophosphamide i.p.i. (Full chemo) and 48 h later, mice were imaged. C, chemotherapy dose effects on biodistribution of ^111^In-DOTA-DAB4 72 h post-chemotherapy. Mice bearing size-matched EL4 tumors were treated as described in *B*, given i.v. ^111^In-DOTA-DAB4 24 h after chemotherapy, and 48 h later, killed for gamma counting of tumors and organs (n = 3/group). Accumulation of DAB4 is shown as mean %ID/g (±SEM). As indicated, Chemo induced significant dose-dependent tumor accumulation of ^111^In-DOTA-DAB4 (*P*<0.001 versus Control). *Inset*, graph depicts mean counts/pixel (±SEM) after region of interest (ROI) analysis of the imaged mice (n = 3/group). ROI were drawn around tumors, and the gamma camera-detected counts, which were obtained using image acquisition software, were divided by the ROI area measured in pixels; **, *P*<0.01, ***, *P*<0.001 versus other treatments as indicated. D, biodistribution of ^111^In-DOTA-DAB4 and ^111^In-DOTA-Sal5 72 h post-chemotherapy. EL4 tumor-bearing mice (n = 3/group) were given i.v. ^111^In-labeled Sal5-DOTA or DAB4-DOTA without chemo (Control) or 24 h after Full chemo. Gamma radioactivity was imaged as described in *B*, and counted as described in *C*, 48 h after radioligand administration. *Inset*, representative clinical gamma camera images (arrow, tumor; asterisk, central blood pool). Tumor accumulation of DAB4 was antigen-specific irrespective of Chemo use (p<0.001 versus Sal5).

The tumor response to chemotherapy was evaluated using γ-scintigraphy of the radiotracer in untreated mice and mice treated with half- or full-dose chemotherapy. To simulate a clinical scenario, tumor growth was staggered to match tumor sizes (Figure 4A) so that mean values for tumor volume or weight among the experimental groups were not significantly different. As clinical gamma camera images of size-matched tumors of control and treated mice show in Figure 4B, tumor uptake of ^111^In-DOTA-DAB4 (given 24 hours after chemotherapy) increased as the chemotherapy dose increased. These visual data correlated both with the quantitative biodistribution data (Figure 4C) and the semi-quantitative planar estimate of radioactivity (inset, Figure 4C). Chemotherapy-induced tumor targeting of ^111^In-DOTA-DAB4 was not a passive phenomenon because both γ-scintigraphy and γ-counting showed that the isotype control, ^111^In-DOTA-Sal5, accumulated significantly less in tumors irrespective of the use of chemotherapy (Figure 4D).

### Comparisons of indices of tumor cell apoptosis with tumor accumulation of ^111^In-DOTA-DAB4 and survival of EL tumor-bearing mice

**Figure 5:**
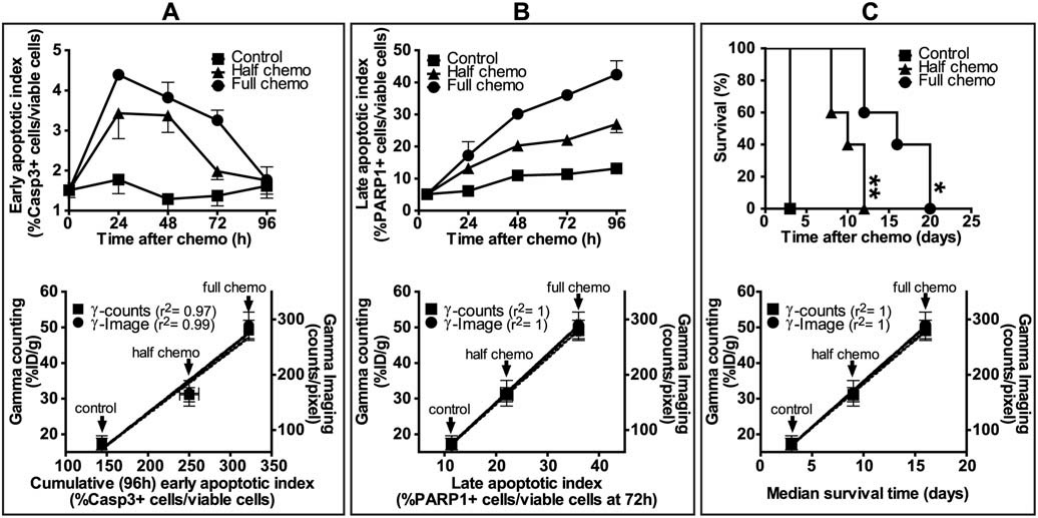
Graphical comparisons of apoptotic indices, tumor accumulation of DAB4, and survival of tumor-bearing mice. EL4 tumor-bearing mice were untreated (Control) or treated with half- or full-dose chemotherapy. Early and late apoptotic cells were identified in tumor sections by staining for activated caspase-3 and cleaved PARP-1, respectively. Using phase color analysis, apoptotic cells were counted and expressed as a percentage of viable cell number to create an apoptotic index. Upper row of panels show time courses of A, early apoptotic index, and B, late apoptotic index, or C, Kaplan-Meier survival analysis of control mice and treated mice (** *P*<0.01 for half-dose chemotherapy versus control, * *P*<0.05 half-dose chemotherapy versus full-dose chemotherapy). In the lower row of panels, peak tumor accumulation of DAB4, which was measured using either gamma counting (%ID/g) or gamma camera imaging (counts/pixel) 72 h post-chemotherapy, was plotted as a function of *A*, the cumulative early apoptotic index, which represented mean %(number of activated caspase-3^+^ cells×h)/(number of viable cells)±SEM (n = 3/group), or *B*, the late apoptotic index, which represented mean %(number of PARP-1^+^ cells)/(number of viable cells)±SEM at 72 h (n = 3/group), or *C*, the median survival time (n = 5/group). Regression coefficients (*r^2^*) for fitted lines are displayed in brackets next to each curve. Chemotherapy-induced tumor apoptosis correlated with both tumor accumulation of DAB4 and survival of tumor-bearing mice.

Immunohistochemical and phase color analyses of tumor sections (Figure 3) were used to derive indices of early or late apoptosis (Figure 5) and of necrosis (see Figure S3 online). After full-dose chemotherapy, the early apoptotic index marked by caspase-3 activation peaked at 4.5%, 24 hours post-chemotherapy (upper panel, Figure 5A), the late apoptotic index marked by PARP-1 cleavage peaked at 35–40%, 48–96 hours post-chemotherapy (upper panel, Figure 5B), whereas the necrotic index remained at 15–20% during the same 48–96 hour period (see Figure S3 online). As caspase-3 activation is early and transient, a cumulative early apoptotic index was determined as the area under the curve generated from results of individual caspase-3 activation assays. Peak EL4 tumor accumulation of ^111^In-DOTA-DAB4 (at 72 hours post-chemotherapy), which was measured as imaged or counted gamma radioactivity, correlated directly with both the cumulative early apoptotic (lower panel, Figure 5A) and late apoptotic indices (lower panel, Figure 5B) but correlated inversely with the necrotic index (see Figure S3 online). The survival of EL4-tumor bearing mice also directly depended on chemotherapy dose so that doubling the dose of chemotherapy significantly extended the survival of tumor-bearing mice (Figure 5C upper panel). Moreover, just as tumor gamma counts from ^111^In-DOTA-DAB4 accumulation correlated directly with indices of apoptosis so the counts correlated directly with the median survival time of treated mice (lower panel, Figure 5C).

### Gamma-scintigraphic detection of tumor apoptosis in vivo using fragments of DAB4

**Figure 6:**
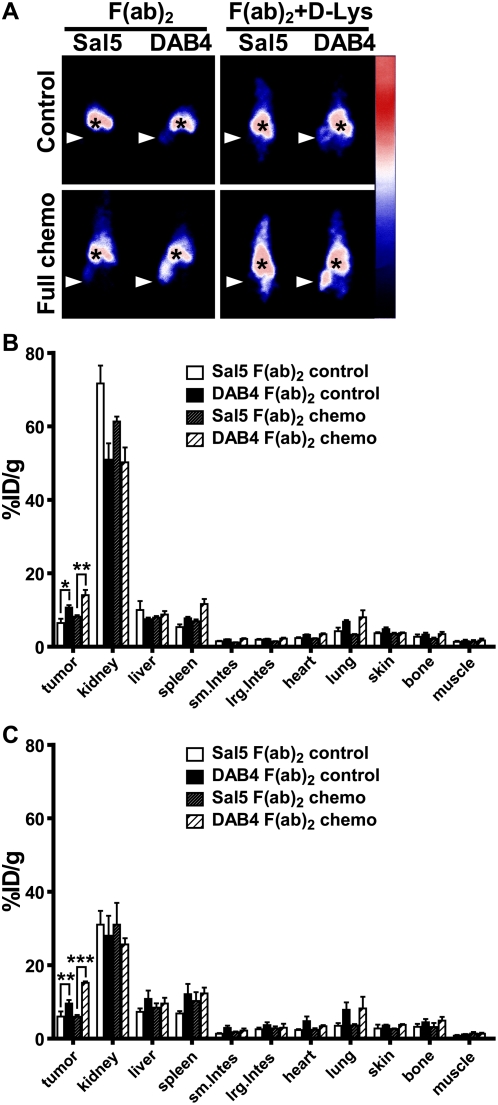
Imaging and biodistribution of antibody fragments. DOTA-conjugated F(ab)_2_ fragments of Sal5 or DAB4 were ^111^In-labeled and given i.v.i. to untreated (control) or treated EL4 tumor-bearing mice 24 h after full-dose chemotherapy. Some mice receiving F(ab)_2_ fragments also received regular D-lysine injections. Mice were imaged with a gamma camera 24 h after radioligand administration, and then killed to measure gamma counts in tumors and normal organs. A, shown are representative gamma camera images of n = 3 mice. Accumulation of radioligand is shown as mean %ID/g±SEM (n = 3/group) for F(ab)_2_ forms of Sal5 and DAB4 B, without or C, with co-injection of D-lysine at 24 h (* *P*<0.05, ** *P*<0.01, *** *P*<0.001 for DAB4 versus Sal5). Antigen-specific and chemotherapy-dependent tumor accumulation of DAB4 occurred independently of the Fc fragment.

Antibody fragments are often used as imaging probes because washout of the probe is sufficiently slow to allow tumor accumulation but rapid enough to provide an adequate tumor to background ratio [36]. Accordingly, our earlier studies demonstrated that the ^111^In-labeled F(ab)_2_ fragment of the La-specific mAb generated a higher tumor to blood ratio than the full-length mAb because of its faster blood clearance. As shown in Figure 6, the response of EL4 tumors to chemotherapy was specifically detected using DAB4-F(ab)_2_, and the co-injection of D-lysine significantly lowered renal uptake. Static images indicate that tumor uptake of the F(ab)_2_ fragment was antigen-specific and increased by co-administration of D-lysine, which reduces renal tubular reabsorption of antibody fragments (Figure 6A). These data were confirmed by measurement of gamma counts in an organ assay (Figure 6B&C).

## Discussion

Our results indicate that the DAB4 mAb, which binds the RNA-binding protein La/SSB, accumulates selectively in tumors after tumor-bearing mice are treated with cytotoxic chemotherapy. We have previously proposed that nuclear RNA-binding proteins contained within apoptotic cancer cells may be useful molecular targets for cancer diagnosis or therapy [29]. This hypothesis is based on the abundance and overexpression of this class of protein in malignant cells as well as their retention and accessibility during apoptosis [37]–[39]. In vitro studies showed that the ubiquitous and exceedingly abundant ribonucleoprotein, La/SSB [40], which is essential for early mammalian development [41], has properties that distinguish it from other cytoplasmic and nuclear antigens during the malignant cell death caused by DNA-damaging treatment [29]. La acts as a molecular chaperone for transfer and ribosomal RNA species generated by RNA polymerase III, which is overactive in malignancy [42]. In addition, La may be involved in coupling of transcription and translation [43]. We discovered that La is overexpressed in malignancy and actively induced in apoptotic malignant cells in response to DNA-damaging treatment [29]. La overexpression in malignancy is consistent with the increased ribosomal biogenesis and protein synthesis that are invariant features of malignancy [44], and associated with overexpression of RNP in the nucleolus, the ribosome factory of the cell [45].

Other properties of the La antigen may also help to explain its identification as a specific marker of tumor apoptotic response. During DNA-damaging treatments such as cisplatin [29] and γ-radiation (Al-Ejeh et al., unpublished observations), higher levels of La antigen are detected and co-localize with the γH2AX marker of DNA double-stranded break formation in malignant cells in vitro [29], suggesting that La is involved in early DNA-damage responses. Interestingly, in support of the claim that La-specific antibody detects cells dying as a result of DNA-damage, immunofluorescence was not a sensitive enough technique to detect binding of this La-specific mAb to spontaneously apoptotic LNCaP prostatic carcinoma cells in vivo [46]. In contrast, we found that La-specific mAb specifically accumulates in DNA-damaged and dying human cancer cell lines in a time- and dose-dependent manner. Lastly, La antigen is crosslinked in dead malignant cells by transglutaminase 2, and this protein crosslinking extends to include fixation of the La-bound mAb itself so that its levels are higher than in dead primary cells [29].

The EL4 lymphoma model was chosen because cyclophosphamide/etoposide treatment induces robust tumor apoptosis [34]. The results from our kinetic studies indicate that chemotherapy itself did not affect the blood clearance half-life of ^111^In-DOTA-DAB4 in tumor-bearing mice. Therefore, it is unlikely that chemotherapy resulted in significant shedding of the DAB4 target antigen into the blood of tumor-bearing mice. However, the administration of ^111^In-DOTA-DAB4 24 hours after chemotherapy did significantly accelerate the rate of DAB4 tumor accumulation, which may have resulted in the enhanced clearance of DAB4 from blood and normal organs. The increased tumor binding of ^111^In-DOTA-DAB4 is proposed to have resulted from a significant increase in the number and strength of DAB4 binding targets, which was related to both the post-chemotherapy number of dead tumor cells as well as the extent to which the La autoantigen was induced in each dying cell.

The immunohistologic analyses defined the dynamics of the apoptotic response of EL4 tumors to chemotherapy. Caspase-3 activation occurred within the first 48 hours of treatment, and peaked 24 hours post-chemotherapy. Subsequently, the level of caspase-3-mediated cleavage of PARP-1 reached a maximum 96 hours post-chemotherapy, which is when ex vivo binding of DAB4 to dead EL4 cells was maximal.

Both caspase-3 activation and PARP-1 cleavage as early and late markers of apoptosis, respectively, increased directly as a function of chemotherapy dose. However, after full-dose chemotherapy, fewer than 5% of tumor cells contained activated caspase-3, whereas cleaved PARP-1 was detected in up to 40% of tumor cells. Similarly, the time-dependent cleavage of PARP-1 followed the rapid and transient activation of caspase-3 after cytotoxic drug treatment or ionizing radiation in vitro (data not shown). These studies illustrate the difficulty of capturing in vivo the dynamic elements of the apoptotic process such as caspase-3 activation, a difficulty that is manifest clinically. For example, frequent invasive sampling of tumor tissue was required to demonstrate that increases in apoptotic index predicted pathologic responses of breast cancer to neoadjuvant chemotherapy [25], [26].

Scintigraphic imaging of control and treated mice indicated that tumor accumulation of ^111^In-DOTA-DAB4 was influenced by the chemotherapy regimen in a linear dose-dependent manner. In stark contrast, the chemotherapy had no significant effect on the radioligand distribution to harvested organs. After chemotherapy, although the significantly greater tumor accumulation of ^111^In-DOTA-DAB4 than ^111^In-DOTA-Sal5 indicated the antigen-specific nature of antibody tumor targeting, twice as much of the injected dose of ^111^In-DOTA-Sal5 accumulated in the tumors of treated mice than untreated mice. However, similar observations were not made for the F(ab)_2_ fragments of Sal5, which suggests that the non-specific tumor accumulation of Sal5 IgG after chemotherapy may be Fc-dependent.

Tumor accumulation of DAB4 was linearly related to the increase in cleaved PARP-1 within EL4 tumors. Furthermore, biotinylated DAB4 injected into treated mice localized best in EL4 cell remnants associated with cleaved PARP-1. Given that tumor accumulation of DAB4 most directly reflects pathologic markers of late rather than early apoptosis, we argue that ^111^In-DOTA-DAB4 is a specific radioligand to detect the enduring tumor response to effective DNA-damaging chemotherapy.

The results also demonstrate that the highest dose of chemotherapy resulted in the highest tumor accumulation of radioligand, and this dose ultimately extended the survival of tumor-bearing mice. Therefore, we infer that a diagnostic radioligand such as ^111^In-DOTA-DAB4 can predict the survival of tumor-bearing mice after DNA-damaging and apoptosis-inducing chemotherapy.

Finally, as useful imaging agents require a high tumor to background ratio, we conducted scintigraphic and organ assays of ^111^In-labeled F(ab)_2_ fragments of DOTA-DAB4. Whole IgG versions of radioligands tend to have protracted blood pool activity and reduced tissue penetration whereas the shorter circulatory half life and increased tissue penetration of antibody fragments may produce more effective imaging agents. The results indicate that although tumor accumulation of F(ab)_2_ fragments of DAB4 was both antigen specific and chemotherapy dose-dependent, the circulatory half life, residence time in normal organs, and absolute levels of tumor accumulation of radiolabeled DAB4 fragments all significantly diminished in comparison with the parental DAB4 mAb. Nevertheless, protein engineering techniques together with modifications in the schedule of radioligand administration may be used to optimize the imaging performance characteristics of DAB4 fragments and ensure efficient therapy monitoring.

We found that giving ^111^In-DOTA-DAB4 24 hours after chemotherapy significantly reduced its normal organ accumulation. This observation may be explained by the enhanced tumor uptake of ^111^In-DOTA-DAB4 seen 24 hours after chemotherapy resulting in its accelerated blood clearance, and thus the diminished blood pool of ^111^In-DOTA-DAB4 available to bathe normal organs. Hence, we believe that scheduling the injection of ^111^In-labeled DAB4 fragments at least 24 hours after cytotoxic chemotherapy maximizes the opportunity to obtain an effective radioimmunoscintigraphic agent with a high tumor to background ratio because the target for DAB4 binding may be revealed differentially among the apoptotic cells of normal and malignant tissues. As was discovered in the jejunal apoptosis model, chemotherapy-induced apoptosis in normal tissues runs a shorter (24–36 hour) time course [47] than the several days (at least) apparent in malignant tissues [21]. Differences in the time course of chemotherapy-induced apoptosis between normal and malignant tissues may happen in part because phagocytosis of apoptotic cells is *efficient* in normal tissues [48] but *inefficient* in malignant tissues because an excess of apoptotic cells seems to overwhelm clearance mechanisms [23].

In vivo measurements of cell death, proliferation, metabolism and hypoxia in a multi-parametric design may hold higher prognostic and predictive value than single measurements and could provide surrogates for clinically meaningful endpoints [2]. Ideal cancer therapy monitoring technologies are rapid, robust, and minimally invasive. They reflect tumor heterogeneity, and yield quantitative real-time data about biologically significant processes like apoptosis.

In conclusion, as a late apoptotic malignant cell-death ligand, the La-specific DAB4 mAb detected the heightened and enduring tumor cell-death response resulting from DNA-damaging treatment. Thus, these preclinical data encourage future development of clinically applicable imaging methods for the detection of DNA damage-related tumor cell death arising from treatments such as cytotoxic chemotherapy and ionizing radiation.

## Supporting Information

Figure S1 Delaying the injection until 24 hours after chemotherapy accelerated the blood clearance and tumor accumulation of the radioligand. B6 mice bearing syngeneic EL4 tumors were untreated or treated with full-dose chemotherapy, and given intravenous injections of ^111^In-DOTA-DAB4 alone (clear columns), immediately after chemotherapy (striped columns), or 24 h after chemotherapy (filled columns). Mice were killed at A, 3, B, 24, C, 48, and D, 72 h after injection of ^111^In-DOTA-DAB4 to measure its accumulation in blood, normal organs and tumors, which was calculated as the percentage of mass-normalized counts per minute (cpm) to total cpm of the injected dose at time 0 (%ID/g). Data shown are mean %ID/g±SEM (n = 5), * P<0.05, ** P<0.01, and *** P<0.001 versus control mice (2-way ANOVA).
(0.05 MB DOC)

Figure S2 Expression of La in dead cells in EL4 tumors correlated with the accumulation of DAB4 in vivo. Single tumor cell suspensions from untreated mice were stained with A, 7-AAD alone or B, 7-AAD and Sal5 or DAB4 to determine the specific binding of DAB4 to dead cells. In a separate study, accumulation of DAB4 mAb itself within tumors in vivo was calculated using values for ^111^In-DOTA-DAB4 accumulation after correction for physical radio-decay of Indium-111. Tumor accumulation of DAB4 as %ID/g was plotted against A, tumor cell death measured using 7-AAD uptake or B, the ex vivo binding of DAB4 to 7-AAD^+^ dead cells.
(0.03 MB DOC)

Figure S3 Accumulation of ^111^In-DOTA-DAB4 correlated inversely with tumor necrosis. EL4 tumor sections from untreated (control) and treated mice were stained with H&E and phase color analysis used to determine regions of necrosis. A, Average necrotic index (±SEM, n = 3) was calculated as the percentage of necrotic areas to viable areas at 0, 24, 48, 72, and 96 h. B, Tumor accumulation of ^111^In-DOTA-DAB4 was measured using gamma counting (%ID/g) or gamma camera imaging (counts/pixel) and plotted as a function of the necrotic index at 72 h.
(0.03 MB DOC)
