# Supplementary material for: APOMAB®, a La-Specific Monoclonal Antibody, Detects the Apoptotic Tumor Response to Life-Prolonging and DNA-Damaging Chemotherapy
Source: PLoS One. 2009 Feb 27;4(2):e4558. doi: 10.1371/journal.pone.0004558 (PMC2645692; doi:10.1371/journal.pone.0004558)
Supplement: Supplementary file 1 [file pone.0004558.s001.doc]

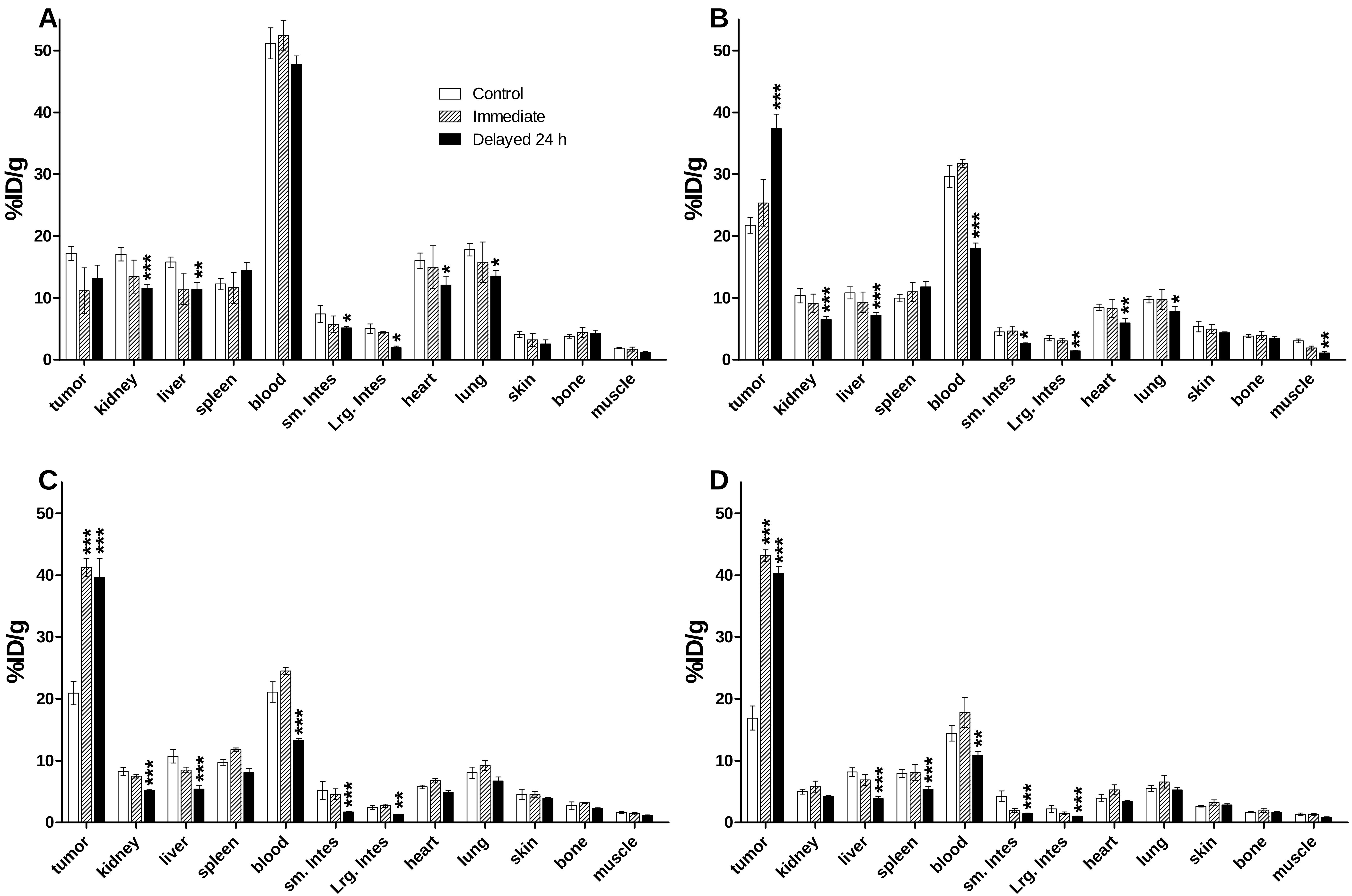

A
B
C
D
%ID/g
Control
Immediate
Delayed 24 h
tumor
kidney
liver
spleen
blood
sm. Intes
Lrg. Intes
heart
lung
skin
bone
muscle
