# Supplementary material for: APOMAB®, a La-Specific Monoclonal Antibody, Detects the Apoptotic Tumor Response to Life-Prolonging and DNA-Damaging Chemotherapy
Source: PLoS One. 2009 Feb 27;4(2):e4558. doi: 10.1371/journal.pone.0004558 (PMC2645692; doi:10.1371/journal.pone.0004558)
Supplement: Supplementary file 2 [file pone.0004558.s002.doc]

A

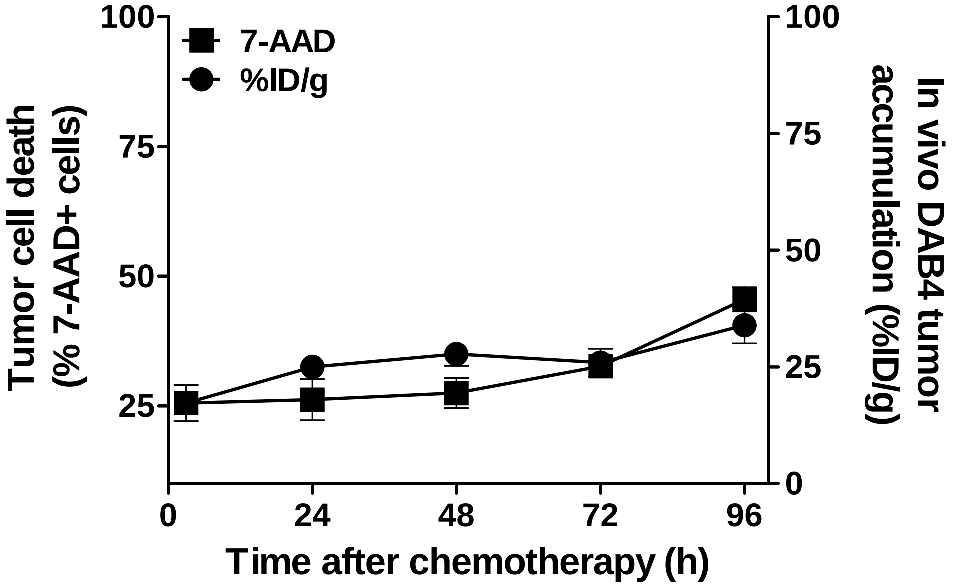
7-AAD
%ID/g
Tumor cell death
(% 7-AAD+ cells)
In vivo DAB4 tumor
accumulation (%ID/g)
100
75
50
25
0
24
48
72
96
Time after chemotherapy (h)

B

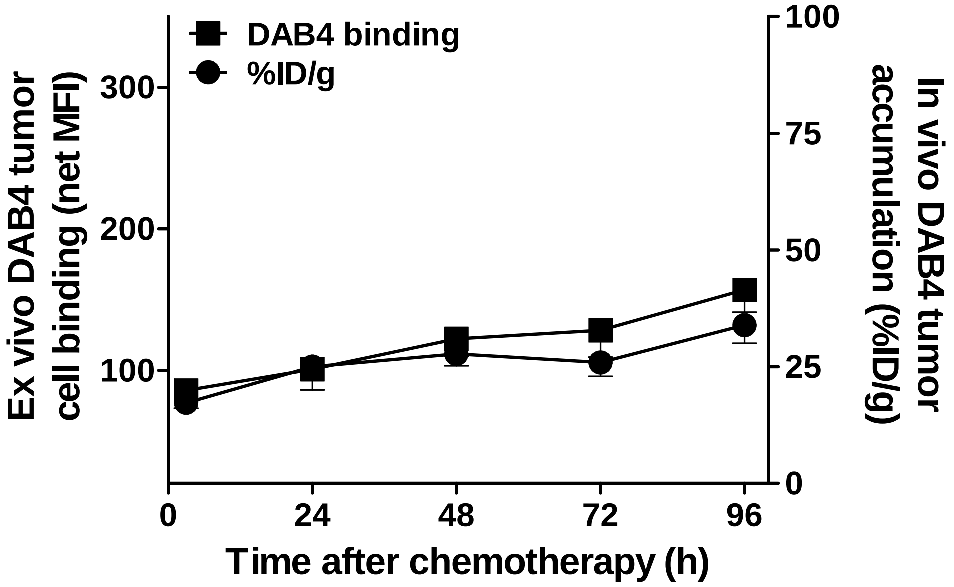
DAB4 binding
%ID/g
Ex vivo DAB4 tumor
cell binding (net MFI)
In vivo DAB4 tumor
accumulation (%ID/g)
300
200
100
75
50
25
0
24
48
72
96
Time after chemotherapy (h)
