# Supplementary material for: APOMAB®, a La-Specific Monoclonal Antibody, Detects the Apoptotic Tumor Response to Life-Prolonging and DNA-Damaging Chemotherapy
Source: PLoS One. 2009 Feb 27;4(2):e4558. doi: 10.1371/journal.pone.0004558 (PMC2645692; doi:10.1371/journal.pone.0004558)
Supplement: Supplementary file 3 [file pone.0004558.s003.doc]

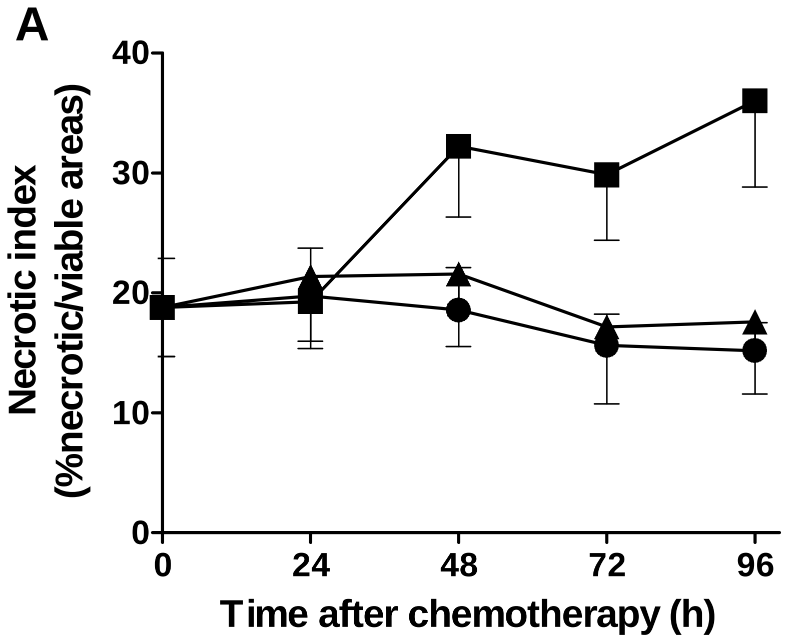
A
Necrotic index
(%necrotic/viable areas)
0
10
20
30
40
0
24
48
72
96
Time after chemotherapy (h)

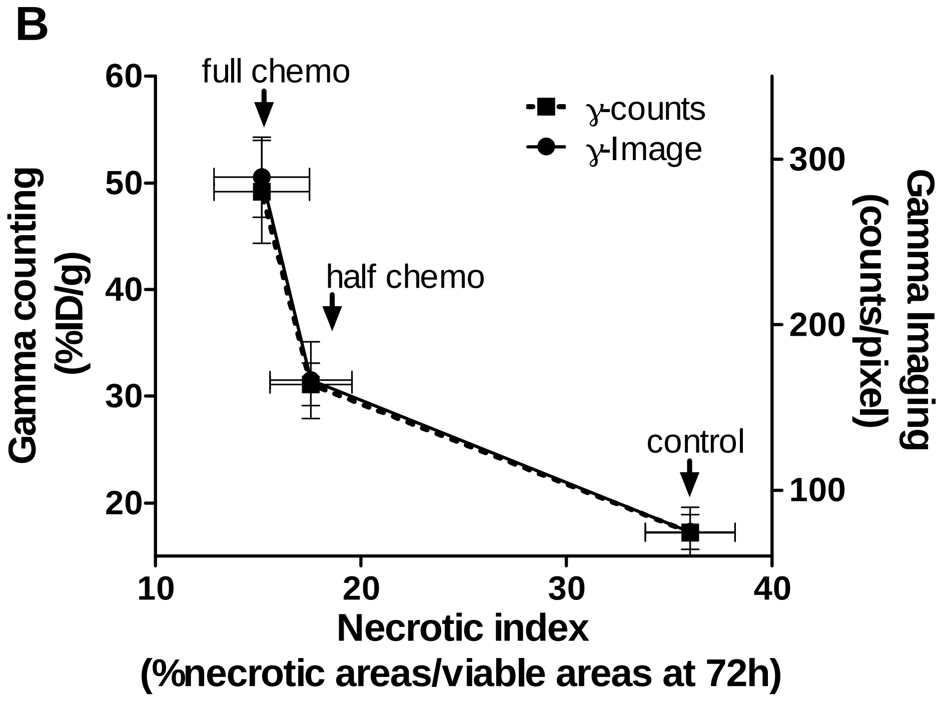
B
full chemo
half chemo
control
γ-counts
γ-Image
Gamma counting
(%ID/g)
20
30
40
50
60
Gamma Imaging
(counts/pixel)
100
200
300
10
20
30
40
Necrotic index
(%necrotic areas/viable areas at 72h)
